# Maternal Circulating Lipid Profile during Early Pregnancy: Racial/Ethnic Differences and Association with Spontaneous Preterm Delivery

**DOI:** 10.3390/nu9010019

**Published:** 2017-01-01

**Authors:** Xinhua Chen, Theresa O. Scholl, Thomas P. Stein, Robert A. Steer, Keith P. Williams

**Affiliations:** 1 Department of Obstetrics/Gynecology, School of Osteopathic Medicine, Rowan University, Stratford, NJ 08084, USA; schollto@rowan.edu (T.O.S.); williakp@rowan.edu (K.P.W.); 2 Department of Surgery, School of Osteopathic Medicine, Rowan University, Stratford, NJ 08084, USA; tpstein@rowan.edu; 3 Department of Psychiatry, School of Osteopathic Medicine, Rowan University, Stratford, NJ 08084, USA; steer@rowan.edu

**Keywords:** spontaneous preterm delivery, serum lipid levels, high-density lipoprotein cholesterol, apolipoprotein A1, racial/ethnic disparity

## Abstract

Prior reports on the association between altered maternal serum lipid levels with preterm delivery are inconsistent. Ethnic differences in serum lipids during pregnancy and their relation to preterm delivery have not been studied. We examined the relationships of six maternal lipids during early pregnancy with the risk of spontaneous preterm delivery (SPTD). The design represents a case-control study nested within a large prospective, multiethnic cohort of young, generally healthy pregnant women. SPTD cases (*n* = 183) and controls who delivered at term (*n* = 376) were included. SPTD is defined as delivery at <37 completed weeks of gestation without indicated conditions. We found that African-American women had significantly increased levels of high-density lipoprotein cholesterol (HDL-C) and apolipoprotein A1 (apoA1), and lower triglyceride (TG) and apolipoprotein B (apoB) levels compared to Hispanic and non-Hispanic Caucasians combined. Elevated HDL-C and apoA1 concentrations were significantly associated with an increased odds of SPTD after controlling for potential confounding factors. The adjusted odds ratio (AOR) was 1.91 (95% confidence interval (CI) 1.15, 3.20) for the highest quartile of HDL-C relative to the lowest quartile, and for apoA1 the AOR was 1.94 (95% CI 1.16, 3.24). When controlling for ethnicity, the results remained comparable. These data suggest that pregnant African-American women had a more favorable lipid profile suggestive of a reduction in cardiovascular risk. Despite this, increased HDL-C and apoA1 were both found to be associated with SPTD.

## 1. Introduction

Preterm delivery (<37 weeks gestation) occurs in 10%–12% of all births in the USA and is a serious health problem for both mother and offspring [1]. African-American women are 50%–60% more likely to deliver a preterm infant than Caucasian women [2]. Apart from medically indicated preterm delivery, the specific causes and mechanisms for spontaneous preterm delivery (SPTD) remain largely unknown [3].

Maternal circulating cholesterols and free fatty acids (FFAs) are important substrates for both mother and the fetus during pregnancy [4,5]. Although the relationship of maternal lipids to preterm birth has been studied, results are inconclusive [6,7,8,9,10]. Maternal serum total cholesterol (TC) concentration, both high and low, has been associated with an increased risk of preterm delivery (PTD) [7,9,10]. Elevated maternal triglycerides (TG) as well as a small change in TG level from preconception to pregnancy have been associated with ~2- to 3-fold increases in risk for PTD [6,8,9,10]. Other studies, however, have found no relation [7,11]. Although the effects of maternal high-density lipoprotein cholesterol (HDL-C), a traditional cardioprotective marker, have been explored [9,10], only Catov et al. reported higher HDL-C to carry an excess risk of preterm birth in parous women [7]. In another case-control study, women with simultaneously increased serum FFAs and HDL-C also showed a greater risk of spontaneous preterm birth [12]. The mechanisms whereby altered maternal lipid levels, especially increases in good cholesterol (HDL-C), might lead to preterm delivery are not understood [7,12]. We are unaware of any reports about the relationships of apolipoproteins A1 and B (apoA1 and apoB)—which are sometimes viewed as sensitive markers of subsequent cardiovascular risk—with preterm birth [13,14]. 

Ethnic differences in lipids also have been observed; African-American men and nonpregnant African-American women have a more favorable cardiovascular lipid profile compared to Caucasians [15,16]. Similar results were observed in pregnant women from the Netherlands [17]. However, data are unavailable for pregnant women in the U.S.

Thus, we examined the associations of lipid concentrations during early pregnancy with spontaneous preterm delivery. Our purpose was to determine if altered lipid levels linked to cardiovascular disease (CVD) risk would also predict SPTD. Because there is a well-known ethnic disparity in preterm delivery, we also ascertained whether ethnic differences in maternal lipids exist and alter the subsequent odds of SPTD.

## 2. Materials and Methods

### 2.1. Study Design

We conducted a case-control study nested within the Camden Study, a large prospective cohort of young, generally healthy pregnant women residing in one of the poorest cities in the continental United States [18]. The cohort of study participants enrolled between 1996 and 2006 (African-American 38%, Hispanic 45%, non-Hispanic Caucasian 17%) was recruited from among patients enrolling at the Osborn Family Health Center, Our Lady of Lourdes Medical Center, and St. John the Baptist prenatal clinic in Camden, NJ, USA. The institutional review board at the University of Medicine and Dentistry of New Jersey (now, Rowan University; School of Osteopathic Medicine in 2013) approved the study protocol. Informed written consent was obtained from each volunteer after explanation of the nature and purpose of the study was explained. The inclusion criteria were for the women who had a positive pregnancy test and received prenatal care at ≤20 weeks of gestation. A total of 3.5% of the women who had serious nonobstetric problems (e.g., lupus, type 1 or 2 diabetes, seizure disorders, malignancies, acute or chronic liver disease, drug or alcohol abuse, and psychiatric problems) were not eligible for participating in the study. Eighty percent of the patients who were eligible agreed to participate in this study, and 8.3% of participants dropped out after enrollment due to a move from the area or to an early pregnancy loss. A final total of 2379 participants whose pregnancy culminated in a live birth were used to select cases and controls.

### 2.2. Data and Blood Specimen Collection

Data on socioeconomic, demographic, and lifestyle were obtained by interview at entry to care (on average, at week 14.2 ± 4.5, mean ± SD) and updated at weeks 20 and 28 gestation. Participants were scheduled to see study research assistants before or after their regular prenatal visits. Fasting blood samples (>8 h) collected at the same schedule were centrifuged at 4 °C and stored at −80 °C until assayed. Current analyses were only focused on samples collected at entry to care. The accepted window for drawing the sample was ±2 weeks. If the participant did not appear, our policy was to have staff undertake a home visit to obtain the samples. Ethnicity was self-defined. Body mass index (BMI) was computed based on self-reported pregravid weight and measured height by using a stadiometer at entry to prenatal care (kg/m^2^). Maternal obesity was defined as BMI ≥30. 

Because different levels of dietary macronutrient intake and glycemic parameters may alter lipid levels, we included these data in the analysis. Dietary data were obtained by a 24-h recall of the previous day’s diet—at entry and at weeks 20 and 28 gestation—and processed with databases from the Campbell Institute of Research and Technology (Campbell Soup Company) in Camden, UK, as described previously [19]. The database generates data for more than 80 nutrients using the U.S. Department of Agriculture, Agricultural Research Service, USDA National Nutrient Database for Standard Reference (Release 13, 2000) and the Continuing Survey of Food Intakes by Individuals. 

### 2.3. Definition of Cases and Controls

Preterm delivery is defined as delivery at <37 completed weeks of gestation based upon the last menstrual period (LMP) and confirmed or modified by ultrasound evaluation as described in [20]. Briefly, the length of gestation was estimated from the mother’s LMP and confirmed by a routine first- or second-trimester ultrasound. If the crown–rump length (CRL) measured at or before 12 weeks of gestation was within 7 days of menstrual age for the LMP, or the biparietal diameter (BPD) measured for second trimester was within 10 days of menstrual age, the estimated date of delivery was based on the LMP. Otherwise, if these limits were exceeded, the estimated date of delivery was based on the CRL or BPD. Detailed information identifying women with spontaneous preterm delivery and medically indicated preterm delivery was obtained from the prenatal, labor, delivery, and newborn records. Information on reproductive history, including prior preterm delivery as well as the medical events during the current pregnancy, was also abstracted.

Spontaneous preterm delivery (*n* = 192) was defined by the presence of intact membranes and regular contractions and by the absence of induction of labor or an elective caesarean section. Preterm premature rupture of membrane (PROM) was defined as rupture of membranes before the onset of labor in the spontaneous preterm group. Women with medically indicated preterm delivery (*n* = 48) were excluded from sample selection. The final of 183 spontaneous preterm delivery cases from the underlying cohort studied with available blood samples were included in the analysis. Nine SPTD cases were excluded due to insufficient blood serum sample for lipid measurements. Normal controls (*n* = 376) were randomly selected without matching any maternal characteristic variables from among women who delivered a term infant (≥37 weeks of gestation) using SAS SURVEYSELECT to assure a similar distribution of maternal characteristics in cases and controls. 

### 2.4. Analytic Procedures

Maternal lipid profile including TC, HDL-C, low-density lipoprotein cholesterol (LDL-C), TG, and apoA1 and B were measured. Serum concentrations of TC, HDL-C, LDL-C, and TG were measured by enzymatic assay kits (Wako Chemicals USA, Inc., Richmond, VA, USA) on a microplate reader. ApoA1 and B were determined by commercial enzyme-linked immunosorbent assay (ELISA) (Mabtech, Cincinnati, OH, USA). The coefficient of variation (CV) for intra- and interassay was 3.6% and 5.0% for TC, 1.8% and 4.5% for HDL-C, 2.9% and 5.2% for LDL-C, 5.3% and 10.1% for TG, 4.0% and 4.5% for apoA1, and 5.0% and 6.9% for apoB.

Fasting plasma glucose was measured by the glucose oxidase method (glucose reagent from Sigma Diagnostics, St. Louis, MO, USA) on a spectrophotometer at a wavelength of 505 nm. Serum insulin was determined by radioimmunoassay (RIA) using a kit with a specific antibody that only minimally cross-reacts (<0.2%) with proinsulin and has a high sensitivity (2 μU/mL or 12 pmol/L). Plasma C-peptide was determined by a RIA kit with high sensitivity (0.1 ng/mL or 0.033 nmol/L) and low cross-activity to proinsulin (<4%, Linco, St. Charles, MO, USA). The intra- and interassay CV was 3.2% and 6.3% for C-peptide, 3.5% and 6.5% for insulin, and 1.5% and 3.0% for glucose. All samples were analyzed in duplicate. Homeostatic model assessment for insulin resistance (HOMA IR) was calculated as described in [21]. 

### 2.5. Statistical Analysis

Maternal characteristics between cases and controls were compared by calculating independent t-tests for continuous variables and chi-square (χ^2^) tests for categorical variables. Multiple regression analyses were performed with SAS GLM to assess the significance of linear trends and compare the mean levels of each of the six lipids between the cases and controls. We first compared the mean lipid levels among three ethnic groups within the cases and controls, then combined the Hispanic and non-Hispanic Caucasian groups and compared them to African Americans. The interaction between ethnicity and preterm delivery was assessed for each lipid to confirm the association between maternal lipids and preterm delivery for all of the ethnic groups.

We next divided each lipid concentration into quartiles based on the distribution of the controls. Logistic regression analyses were used to estimate the unadjusted odds ratio (OR), adjusted odds ratio (AOR), and 95% confidence interval (95% CI) for the associations of maternal lipid levels (comparing each quartile to the lowest quartile (as the reference category)) with spontaneous preterm delivery. Separate models were used to examine the relation between each lipid and spontaneous preterm delivery, and the analyses were also stratified by ethnic group. Hosmer–Lemeshow goodness of fit tests (group = 10) were performed for logistic regression models for each lipid to confirm if the model was correctly specified. 

Potential confounding variables were defined as those which altered the adjusted odds ratio or means by at least 10%, and were assessed by comparing crude and adjusted data. Based upon our prior experience with the cohort [20,22,23], these included maternal pre-pregnancy BMI, age, parity, and cigarette smoking, which were controlled for in all of the multivariate regression models. Spontaneous preterm delivery as the outcome variable was coded (0 = No, 1 = Yes) for cases and controls. Dummy variables were created for three ethnic groups (African American, Hispanic, and non-Hispanic Caucasian) and also for the two ethnic groups combined (Hispanic and non-Hispanic Caucasian). The level of significance was set at the 0.05, two-tailed level. All of the statistical analyses were performed using SAS v.9.3 (SAS Institute, Inc., Cary, NC, USA). 

## 3. Results

### 3.1. Clinical Characteristics

Table 1 shows descriptive data on maternal characteristics; parity, cigarette smoking, gestational age at blood sampling, medical insurance, and blood pressure at entry were not significantly different between cases and controls. The cases were older (*p* < 0.01) and with a higher pre-pregnancy BMI (*p* < 0.05) than the controls. As expected, cases had significantly shorter gestations (*p* < 0.0001) and lower infant birth weights (*p* < 0.0001) and more of the cases had a prior history of preterm delivery (*p* < 0.001). No significant difference in prevalence of gestational diabetes and preeclampsia was observed. The proportions of each ethnic group were also not different between cases and controls due to the nature of random selection design (*p* = 0.432).

Maternal dietary macronutrient intake (fat, protein, and carbohydrate), fasting levels of glucose, insulin, C-peptide, and HOMA IR at entry to care were not significantly different (*p* > 0.05 for each) with the exception of marginally elevated level of serum insulin (*p* = 0.054, Table 2) for the cases.

### 3.2. Racial/Ethnic Differences in Maternal Lipid Concentrations

Ethnic differences in maternal lipid levels were tested by case-control status (Table 3). In the cases, African-American and Hispanic women had significantly elevated mean HDL-C levels as compared to the non-Hispanic Caucasians (*p* < 0.05). African Americans also had a decreased TG levels as compared to the non-Hispanic Caucasians (*p* < 0.01). There were no differences in the levels of TC, LDL-C, apoA1, and apoB (*p* > 0.05 for each). In the controls, African-American women had higher concentrations of HDL-C and apoA1, and a lower concentration of TG as compared to either Hispanic or non-Hispanic Caucasian groups (*p* < 0.05 to *p* < 0.001). No significant differences were detected between Hispanic and non-Hispanic Caucasian women. Similar differences were observed when we compared the African American group to the combined Hispanic and non-Hispanic Caucasian group. In addition, we did not find a significant interaction between ethnicity and preterm delivery for any of the lipids (*p* > 0.05 for each).

### 3.3. Maternal Lipid Profile and Spontaneous Preterm Delivery

After controlling for the four confounding variables and adding ethnicity, the mean levels of fasting serum HDL-C and apoA1 were significantly higher in the SPTD cases as compared to the term-delivery controls, whereas the mean levels of the other four lipids were comparable for both groups (*p* > 0.05 for each, Table 4). 

The result of Hosmer–Lemeshow goodness of fit test for logistic regression models for each lipid indicated that models were adequately fitted (*p* > 0.05 for each). The *p*-value was 0.539 for HDL-C model and 0.878 for ApoA1 model. Elevated levels of HDL-C (>75th percentile or ≥1.40 mmol/L) and apoA1 (≥1.26 g/L) were positively associated with the risk of SPTD (Table 5). The AOR was 1.91 (95% CI 1.15, 3.20) (Table 5, model 1) for the highest quartile of HDL-C relative to the lowest quartile; similar results were obtained for apoA1 (AOR was 1.94, 95% CI 1.16, 3.24). With the added inclusion of ethnicity (Table 5, model 2), the results were comparable. No significant relationships were found between TC, LDL, TG, and apoB with SPTD. 

Finally, we performed additional analyses to confirm if the association of high HDL-C and apoA1 with SPTD is the same in each ethnic group. Although the unadjusted proportion of the highest quartile of HDL-C and/or apoA1 in SPTD cases of each ethnic group tended to be higher than in the controls, only high HDL-C (AOR 2.79, 95% CI 1.28, 6.11) in Hispanics and apoA1 in non-Hispanic Caucasians (AOR 6.24, 95% CI 1.44, 27.08) reached statistical significance. However, there was a ≥2-fold increased odds of SPTD as compared to the highest quartile of HDL-C to the lowest quartile when Hispanics and non-Hispanic Caucasians were combined (AOR 2.29, 95% CI 1.16, 4.53) or Hispanics and African Americans (AOR 1.98, 95% CI 1.13,3.46) were combined. Similar results were obtained for high apoA1 with the risk of SPTD (Table 6). All of the analyses were controlled for the same potential confounding variables.

## 4. Discussion

Our purpose was to determine if differences in lipid levels linked to CVD risk were predictive of SPTD. In this prospective, case-control study nested within a large cohort of healthy pregnant women from Camden, NJ, USA, we found two new and clinically important relationships. There were ethnic differences in maternal lipid levels; African-American women had a favorable CVD lipid profile compared to other ethnic groups. We also demonstrated that elevated maternal levels of HDL-C and apoA1- lipids, which are cardiovascular protective, were associated with an increased odds for SPTD, independent of ethnicity and several other traditional risk factors. 

### 4.1. Association of High HDL-C and apoA1 with SPTD

During pregnancy, maternal cholesterol crosses the placenta directly using specialized transporters, from the maternal circulation to the fetus [4,5], whereas triglycerides are first broken down into FFAs before crossing the placenta [24]. Both cholesterol and FFAs are important substrates for fetal growth and development. Cholesterol is essential for the synthesis of fetus cell membranes and a precursor for steroid hormones [4,25]. Maternal lipid concentrations change dramatically during pregnancy; altered maternal lipid levels have [6,7,8] and have not [11] been related to preterm delivery. 

A prior study reported that if a mother’s pre-pregnancy TC is either very low (<25th percentile, 94–155 mg/dL) or very high (>75th percentile, 196–318 mg/dL), then her risk for a preterm birth increases 2- to 5-fold [7]. However, this study was unable to distinguish medically induced PTD from spontaneous PTD [7]. Other researchers have observed that higher TC levels before or during pregnancy were associated with a 1.5- to a 3-fold increased risk for a preterm birth [9,10]. These inconsistent findings may be attributable to the timing of the sample collection for lipids (pre-conception, during pregnancy), clinical presentation and the types of preterm delivery studied (medically indicated vs. spontaneous), or the gestations used as an outcome of interest (e.g., very preterm (<34 weeks) vs. moderate preterm (34–<37 weeks)) [6,7,8,9,10].

As previously mentioned, we are unaware of any prior reports linking maternal apoA1 and apoB with preterm delivery. Our study investigated six lipids, including apoA1 and B. ApoA1 is required for normal HDL biosynthesis and is present virtually in all HDL particles [26]. ApoB is the main protein constituent of very low-density lipoprotein (VLDL) and LDL and is known to be involved in atherosclerosis and cardiovascular disease [13,14,26]. 

In the present study, the SPTD cases had significantly higher mean levels of HDL-C and apoA1 (*p* < 0.001) compared to the controls (Table 4). Elevated HDL-C and apoA1 (>75th percentile) were associated with an approximately 2-fold increases in the risk for delivering a preterm infant (Table 5, model 1). After controlling for ethnicity along with four other established confounders, the adjusted odd ratios remained the same (Table 5, model 2). In contrast to previous studies, we did not find significant associations of TC and TG with SPTD.

Although a decreased HDL-C level during pre-pregnancy or mid-pregnancy has been associated with greater risk of PTD [8,9], other researchers have found no relationship [10]. Our study confirmed the results from two prior studies; Catov et al. reported that parous women with pre-pregnancy HDL-C in the highest quartile had an excess risk of PTD (AOR 4.41, 95% CI 1.56–12.48) [7]. By factor analysis, the same author observed that increased maternal FFAs and HDL-C levels at first trimester had an odds ratio at 1.5 and 1.9 for delivering at 34–36 weeks and <34 weeks, respectively [12].

It is not known why increased HDL-C and apoA1 are associated with an increased risk of SPTD. One of the important functions of HDLs is to accept excess cholesterol from peripheral cells and tissues and transport it back to the liver for disposal into the bile (reverse cholesterol transport), thus reducing cholesterol concentration in the blood stream [26,27]. HDL-C concentration has been a traditional factor to assess cardiovascular risk; increased HDL-C is atheroprotective [26,27]. However, there have been recent challenges to the HDL-C hypothesis suggesting that HDL-C concentration does not always reflect its functionality [28,29]. Although the HDL-C level was correlated with the cholesterol efflux capacity of HDL (an assay to measure HDL function), studies with non-pregnant subjects have found that the cholesterol efflux capacity of HDL was inversely associated with CVD events independent of HDL-C concentration [28,29]. 

The current study was not designed to demonstrate mechanisms. However, based on the knowledge that many factors regulate HDL synthesis and/or function including genotype or polymorphism, multiple enzymes (such as cholesterol transport protein), oxidative stress, and inflammatory response [26,30], we hypothesize that increased HDL-C may be a biomarker of oxidative stress and proinflammation, which are known contributors to preterm delivery [2,3,22,23]. More studies are needed to confirm our findings, to explore if and to what extent there is HDL dysfunction, and whether other regulatory factors contribute to HDL dysfunction in women with SPTD.

### 4.2. Ethnic Differences in Maternal Lipids and SPTD

Information on ethnic differences in the lipid profile during pregnancy is limited. A single report from the Netherlands suggested that African-Caribbean women had higher HDL-C, and lower serum TC, LDL-C, apoB, and TG as compared to Dutch women during early and late gestation [17]. Our findings are consistent; in the U.S., African-American women, both cases and controls, had a lipid profile that also was favorable to cardiovascular protection (i.e., higher levels of HDL-C and apoA1, lower TG and apoB (in controls only)) when compared to other ethnic groups (Table 3). These data are consistent with findings from non-pregnant subjects [16,31,32]. However, the underlying mechanisms for these differences are unclear.

We also investigated if ethnic differences in maternal lipids influence risk of SPTD and found no significant interactions between ethnicity and preterm delivery for the six lipids that were studied, suggesting that the associations between HDL-C and apoA1 with the risk of SPTD are the same in each ethnic group, not just in the African-American group. In addition to controlling for several traditional risk factors, we also controlled for ethnicity in both continuous and logistic multiple regression analyses (Table 4 and Table 5), and the relationships of HDL-C and apoA1 with SPTD were consistently comparable. Additional analyses stratified by ethnic group showed a similar trend; there was a greater than 2-fold increased risk for SPTD when the Hispanics were combined with either the non-Hispanic Caucasians or African Americans (Table 6). These results suggest that the associations of high HDL-C and apoA1 with SPTD are independent of maternal ethnicity. 

African-American women have greater risk for preterm delivery than other ethnic groups [2]. Our data did not provide evidence that ethnic disparities in serum lipid levels further increased their risk (Table 6). A possible explanation is this lack of evidence may be attributable to the study’s design. By random selection, several maternal characteristics were uniformly distributed in the cases and controls, including the proportion of each ethnic group (*p* = 0.432, Table 1). There were also only 27 non-Hispanic Caucasian SPTD cases included in this nested case-control study, which resulted in insufficient statistical power to detect significant differences between them and the other ethnic groups. However, the present study’s ethnic distribution does match that for Camden City—a city with the highest poverty rates. One of our goals was to study the outcomes of pregnancy for women living in the Camden City [18]. With a larger prospective cohort and different ethnic distribution, the effect of ethnic disparities in lipids may become more pronounced. 

## 5. Conclusions

Our research supports that there are ethnic differences in maternal lipid levels; African-American women had a more favorable lipid profile suggestive of increased cardiovascular protection. The new findings of elevated HDL-C and apoA1 being associated with risk for SPTD independent of ethnicity and several other traditional risk factors are surprising, and these data underscore the need for further research linking HDL-C dysfunction and other regulatory factors of HDL-C in women with SPTD. 

## Figures and Tables

**Table 1:** Clinical characteristics of spontaneous preterm delivery cases and term controls ^a^.

Variable	Cases	Controls	*p*-Value
	*n* = 183	*n* = 376	
Age (years)	23.08 ± 6.19	21.48 ± 4.92	0.001
Body mass index (BMI) (kg/m^2^)	26.35 ± 6.50	25.16 ± 5.42	0.025
Obesity (BMI ≥30) *n* (%)	43 (23.50)	60 (15.96)	0.031
Nulliparas *n* (%)	67 (36.61)	158 (42.02)	0.221
Cigarette smoking *n* (%)	45 (24.59)	71 (18.88)	0.118
Ethnicity *n* (%)			
Hispanic	86 (46.99)	178 (47.34)	
African American	70 (38.25)	128 (34.04)	
Non-Hispanic Caucasian and other ^b^	27 (14.75)	70 (18.62)	0.432
Medicaid *n* (%)	181 (98.91)	366 (97.34)	0.454
Enrollment duration (years)	5.63 ± 2.67	5.25 ± 2.77	0.113
Blood sample storage before assayed (years)	9.30 ± 2.67	9.00 ± 2.78	0.241
Gestational age at blood sampling (weeks)	16.58 ± 4.86	16.98 ± 5.59	0.415
Blood pressure at entry (mmHg)			
Systolic blood pressure	113.19 ± 14.36	111.52 ± 11.33	0.143
Diastolic blood pressure	70.84 ± 10.27	69.75 ± 8.75	0.197
Prior history of preterm delivery *n* (%) ^c^	28 (24.10)	20 (9.17)	0.0002
Gestational age at delivery (weeks)	32.82 ± 3.97	39.32 ± 1.25	<0.0001
Infant birth weight (g)	2131 ± 854	3311 ± 420	<0.0001
Complicated with preeclampsia *n* (%)	21 (11.93)	37 (10.08)	0.514
Complicated with gestational diabetes *n* (%)	9 (4.91)	16 (4.26)	0.608

^a^ Data are mean ± standard deviation or *n* (%). *p*-values are from analysis of variance or chi-square test; ^b^ Five Asian women were included; ^c^ Prior history of preterm delivery in parous women.

**Table 2:** Maternal glucose metabolism parameters and dietary macronutrient intake in spontaneous preterm delivery cases and term controls ^a^.

Variable	Cases	Controls
	*n* = 183	*n* = 376
Dietary nutrients intake (g/day)		
Total fat	82.95 ± 2.72	84.11 ± 1.86
Polyunsaturated fat	14.22 ± 0.59	13.99 ± 0.40
Monounsaturated fat	31.22 ± 1.08	31.66 ± 0.73
Saturated fat	31.09 ± 1.22	31.83 ± 0.84
Protein	88.78 ± 3.10	90.73 ± 2.19
Carbohydrate	282.72 ± 7.68	290.25 ± 5.25
Glucose metabolism variables (fasting)		
Plasma glucose (mmol/L)	4.49 ± 0.09	4.48 ± 0.06
Serum insulin (pmol/L)	181.26 ± 14.86	146.19 ± 10.49
Serum C-peptide (nmol/L)	0.81 ± 0.05	0.73 ± 0.04
HOMA IR	4.74 ± 0.40	4.07 ± 0.28

^a^ Data are mean ± standard error. Dietary nutrients intake were adjusted for total energy intake; other variables (glucose, insulin, C-peptide, and HOMA IR (homeostatic model assessment for insulin resistance)) were adjusted for pre-pregnancy BMI. All results were not statistically significant (*p* > 0.05 for each) except a marginal difference was detected in insulin level (*p* = 0.054).

**Table 3:** Maternal lipid levels in spontaneous preterm delivery cases and term controls and by racial/ethnic groups ^a^.

		Cases of Preterm Delivery			Term Controls			
Lipid Concentration	African American	Hispanic	Non-Hispanic Caucasian	Hispanic & Non-Hispanic Caucasian Combined	African American	Hispanic	Non-Hispanic Caucasian	Hispanic & Non-Hispanic Caucasian Combined
*n*	70	86	27	113	128	178	70	248
HDL-C (mmol/L)	1.348 ± 0.038 ^b,c^	1.272 ± 0.034 ^b^	1.119 ± 0.062	1.238 ± 0.030	1.293 ± 0.028 ^d,e,f^	1.157 ± 0.024	1.147 ± 0.038	1.154 ± 0.020
ApoA1 (g/L)	1.164 ± 0.038	1.116 ± 0.034	1.177 ± 0.063	1.130 ± 0.030	1.114 ± 0.028 ^g,h,i^	1.035 ± 0.024	1.016 ± 0.039	1.030 ± 0.020
Triglyceride (mmol/L)	1.497 ± 0.115 ^b,c^	1.753 ± 0.099	2.075 ± 0.187	1.824 ± 0.088	1.450 ± 0.084 ^d,e,f^	1.869 ± 0.070	1.742 ± 0.112	1.833 ± 0.059
TC (mmol/L)	4.454 ± 0.119	4.420 ± 0.106	4.510 ± 0.197	4.441 ± 0.093	4.312 ± 0.088	4.386 ± 0.074	4.393 ± 0.120	4.388 ± 0.063
LDL-C (mmol/L)	3.166 ± 0.106	3.173 ± 0.095	3.325 ± 0.176	3.207 ± 0.084	3.115 ± 0.079	3.188 ± 0.067	3.207 ± 0.108	3.193 ± 0.056
ApoB (g/L)	0.920 ± 0.032	0.971 ± 0.028	1.032 ± 0.052	0.985 ± 0.025	0.939 ± 0.023 ^i^	1.017 ± 0.020	0.956 ± 0.032	0.999 ± 0.027

^a^ Data are mean ± SE. Models were adjusted for maternal age, pre-pregnancy BMI, parity, and cigarette smoking; ^b^ *p* < 0.05 vs. non-Hispanic Caucasian cases; ^c^ *p* < 0.01 vs. Hispanic and non-Hispanic Caucasian cases combined; ^d^ *p* < 0.001 vs. Hispanic and non-Hispanic Caucasian controls combined; ^e^ *p* < 0.001 vs. Hispanic controls; ^f^ *p* < 0.01 vs. non-Hispanic Caucasian controls; ^g^ *p* < 0.05 vs. Hispanic controls; ^h^ *p* < 0.05 vs. non-Hispanic Caucasian controls; ^i^ *p* < 0.05 vs. Hispanic and non-Hispanic Caucasian controls combined. HDL-C: high-density lipoprotein cholesterol; ApoA1: Apolipoprotein A1; ApoB: Apolipoprotein B; TC: total cholesterol; LDL-C: low-density lipoprotein cholesterol.

**Table 4:** Maternal lipid levels in spontaneous preterm delivery cases and term controls ^a^.

Lipid Concentration	Cases	Controls
***n***	183	376
HDL-C (mmol/L)	1.275 ± 0.024 ^b^	1.204 ± 0.016
ApoA1 (g/L)	1.141 ± 0.024 ^b^	1.059 ± 0.017
Triglyceride (mmol/L)	1.712 ± 0.070	1.701 ± 0.048
TC (mmol/L)	4.448 ± 0.074	4.361 ± 0.051
LDL-C (mmol/L)	3.195 ± 0.066	3.165 ± 0.046
ApoB (g/L)	0.961 ± 0.020	0.979 ± 0.014

^a^ Data are mean ± SE. Models were adjusted for maternal age, pre-pregnancy BMI, parity, cigarette smoking, and ethnicity; ^b^ *p* < 0.001 vs. controls.

**Table 5:** Association of altered maternal HDL-C and apoA1 with spontaneous preterm delivery: comparing the lowest quartile to other quartiles.

Lipids in Quartiles	Cases *n* (%)	Controls *n* (%)	Model 1 AOR (95% CI) ^b^	Model 2 AOR (95% CI) ^c^
**HDL-C (mmol/L) ^a^**				
<1.01	41 (22.4)	98 (26.1)	1.00	1.00
1.01–1.166	42 (23.0)	97 (25.8)	1.07 (0.63, 1.32)	1.07 (0.63, 1.81)
1.19–1.40	42 (23.0)	99 (26.3)	1.00 (0.60, 1.72)	1.00 (0.59, 1.72)
>1.40	58 (31.7)	82 (21.8)	1.91 (1.15, 3.20)	1.86 (1.10, 3.12)
**ApoA1 (g/L) ^a^**				
<0.86	39 (21.3)	99 (26.7)	1.00	1.00
0.86–1.05	46 (25.1)	92 (24.8)	1.24 (0.73, 2.10)	1.25 (0.74, 2.11)
1.06–1.26	36 (19.7)	103 (27.8)	0.88 (0.51, 1.51)	0.85 (0.49, 1.46)
>1.26	62 (33.9)	77 (20.8)	1.94 (1.16, 3.24)	1.92 (1.15, 3.21)
**Triglyceride (mmol/L)**				
<1.05	40 (21.2)	98 (24.9)	1.00	1.00
1.05–1.45	49 (25.9)	99 (25.2)	1.11 (0.66, 1.85)	1.13 (0.68, 1.89)
1.46–2.11	50 (26.5)	98 (24.9)	0.99 (0.59, 1.68)	1.02 (0.61, 1.73)
>2.11	50 (26.5)	98 (24.9)	1.02 (0.61, 1.72)	1.07 (0.63, 1.82)
**TC (mmol/L)**				
<3.73	40 (21.2)	94 (25.0)	1.00	1.00
3.73–4.22	38 (20.8)	94 (25.0)	0.89 (0.51, 1.53)	0.89 (0.52, 1.55)
4.25–4.95	54 (29.5)	94 (25.0)	1.39 (0.83, 2.32)	1.40 (0.84, 2.35)
>4.95	51 (27.9)	94 (25.0)	1.28 (0.76, 2.15)	1.29 (0.77, 2.17)
**LDL-C (mmol/L)**				
<2.59	46 (25.1)	94 (25.0)	1.00	1.00
2.59–3.06	39 (21.3)	94 (25.0)	0.80 (0.47, 1.36)	0.79 (0.47, 1.34)
3.08–3.68	43 (23.5)	94 (25.0)	0.95 (0.56, 1.59)	0.96 (0.57, 1.63)
>3.68	55 (30.1)	94 (25.0)	1.20 (0.73, 1.98)	1.21 (0.73, 1.99)
**ApoB (g/L)**				
<0.78	47 (25.7)	93 (24.7)	1.00	1.00
0.78–0.96	47 (25.7)	93 (24.7)	1.01 (0.61, 1.67)	1.01 (0.61, 1.67)
0.97–1.12	41 (22.4)	92 (24.5)	0.77 (0.45, 1.31)	0.80 (0.47, 1.36)
>1.12	48 (26.2)	94 (25.0)	0.87 (0.52, 1.46)	0.90 (0.54, 1.52)

AOR, adjusted odds ratio; 95% CI, 95% confidence interval; ^a^ *p* for trend was <0.05 for models 1 and 2; ^b^ Models were adjusted for maternal age, pre-pregnancy BMI, parity, medical payment and cigarette smoking; ^c^ Additional adjustment for maternal ethnicity (African American vs. other ethnic groups).

**Table 6:** Association of elevated maternal HDL-C and apoA1 with spontaneous preterm delivery by racial/ethnic group: comparing the lowest quartile to other quartiles.

Lipids in Quartiles	Cases *n* (%)	Controls *n* (%)	AOR (95% CI) ^a^
**African American**			
**HDL-C (mmol/L)**			
<1.01	14 (20.0)	25 (19.5)	1.00
1.01–1.166	15 (21.4)	32 (25.0)	0.88 (0.34, 2.25)
1.19–1.40	13 (18.6)	30 (23.4)	0.80 (0.31, 2.10)
>1.40	28 (40.0)	41 (32.0)	1.26 (0.54, 2.94)
**ApoA1 (g/L)**			
<0.86	16 (22.9)	32 (25.2)	1.00
0.86–1.05	16 (22.9)	29 (22.8)	1.18 (0.46, 3.00)
1.06–1.26	13 (18.6)	35 (27.6)	0.73 (0.28, 1.92)
>1.26	25 (35.7)	31 (24.4)	1.86 (0.77, 4.50)
**Hispanic**			
**HDL-C (mmol/L) ^b^**			
<1.01	17 (19.8)	52 (29.2)	1.00
1.01–1.166	18 (20.9)	48 (26.9)	1.16 (0.53, 2.54)
1.19–1.40	25 (29.1)	47 (26.4)	1.71 (0.80, 3.66)
>1.40	26 (30.2)	31 (17.4)	**2.79 (1.28, 6.11)**
**ApoA1 (g/L)**			
<0.86	17 (19.8)	47 (26.7)	1.00
0.86–1.05	25 (29.1)	44 (25.0)	1.41 (0.66, 3.02)
1.06–1.26	20 (23.3)	48 (27.3)	1.15 (0.53, 2.49)
>1.26	24 (27.9)	37 (21.0)	1.81 (0.84, 3.93)
**non-Hispanic Caucasian ^c^**			
**HDL-C (mmol/L)**			
<1.01	9 (34.6)	20 (30.3)	1.00
1.01–1.166	9 (34.6)	15 (22.7)	1.16 (0.35, 3.81)
1.19–1.40	4 (15.4)	21 (31.8)	0.40 (0.10, 1.60)
>1.40	4 (15.4)	10 (15.5)	0.96 (0.22, 4.24)
**ApoA1 (g/L) ^b^**			
<0.86	6 (23.1)	20 (31.3)	1.00
0.86–1.05	5 (19.2)	17 (26.6)	0.97 (0.23, 4.13)
1.06–1.26	3 (11.5)	18 (28.1)	0.46 (0.09, 2.37)
>1.26	12 (46.2)	9 (14.1)	**6.24 (1.44, 27.08)**
**Hispanic and non-Hispanic Caucasian combined**			
**HDL-C (mmol/L) ^b^**			
<1.01	27 (23.9)	73 (29.4)	1.00
1.01–1.166	27 (23.9)	65 (26.2)	1.11 (0.58, 2.10)
1.19–1.40	29 (25.7)	69 (27.8)	1.21 (0.64, 2.29)
>1.40	30 (26.6)	41 (16.5)	**2.29 (1.16, 4.53)**
ApoA1 (g/L) ^b^			
<0.86	23 (20.4)	67 (27.5)	1.00
0.86–1.05	30 (26.6)	63 (25.8)	1.39 (0.72, 2.69)
1.06–1.26	23 (20.4)	68 (27.8)	0.92 (0.46, 1.82)
>1.26	37 (32.7)	46 (18.9)	**2.41 (1.23, 4.72)**
**Hispanic and African American combined**			
**HDL-C (mmol/L) ^b^**			
<1.01	31 (19.9)	77 (25.2)	1.00
1.01–1.166	33 (21.2)	80 (26.1)	1.01 (0.56, 1.82)
1.19–1.40	38 (24.4)	77 (25.2)	1.22 (0.68, 2.17)
>1.40	54 (34.6)	72 (23.5)	**1.98 (1.13, 3.46)**
**ApoA1 (g/L) ^b^**			
<0.86	33 (21.2)	79 (26.1)	1.00
0.86–1.05	41 (26.3)	73 (24.1)	1.31 (0.71, 2.41)
1.06–1.26	33 (21.2)	83 (27.4)	1.07 (0.57, 1.99)
>1.26	49 (31.4)	68 (22.4)	**2.01 (1.09, 3.70)**

AOR, adjusted odds ratio; 95% CI, 95% confidence interval; ^a^ Models were adjusted for maternal age, pre-pregnancy BMI, parity, medical payment, and cigarette smoking; ^b^ *p* for trend <0.05; ^c^ Five Asian women were excluded.
